# Slowly but Surely: Larger Brains Improve Immature Survival in Primates

**DOI:** 10.1002/ajp.70072

**Published:** 2025-08-29

**Authors:** Zitan Song, Carel P. van Schaik

**Affiliations:** ^1^ Comparative Socioecology Group, Department for the Ecology of Animal Societies Max Planck Institute of Animal Behavior Konstanz Germany; ^2^ Department of Evolutionary Anthropology University of Zurich Switzerland; ^3^ Institute for the Interdisciplinary Study of Language Evolution University of Zurich Zurich Switzerland

**Keywords:** age at first reproduction, life‐history, parental investment, skill acquisition

## Abstract

The high energy costs of brains suggest that a species' current brain size is adaptive. However, although the comparative data for mammals suggest a positive effect on fitness in larger‐brained species because of higher adult survival and thus longer lifespan, it also reveals two negative effects, namely later age at first reproduction owing to slower development and a tendency towards reduced reproductive allocation owing to larger newborns. Here we suggest that what is missing is the positive impact of brain size on immature survival, causally linked to greater parental investment in larger‐brained species. Using long‐term demographic data on natural populations of 18 primate species, we find a strong positive brain size effect on immature survival, which is already apparent during the first year. We suggest this effect is caused by parental protection and provisioning, allowing young to survive better and mature slowly but surely. This survival effect may well be the strongest adaptive benefit of increased brain size. It remains unknown to what extent this effect generalizes to non‐primates.

## Summary

- This study reveals a so far overlooked and surprisingly strong benefit of larger brains: vastly improved immature survival.

## Graphical abstract

Brain size enhances immature survival, likely offsetting the costs of delayed reproduction and reduced fertility—potentially its key adaptive benefit.

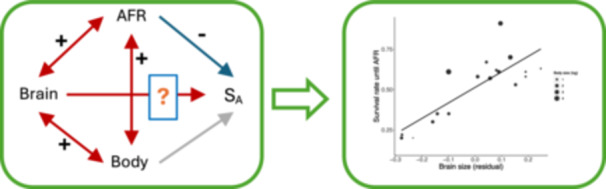

## 1 Introduction

There are various reasons to assume that a species' brain size is an adaptive trait that can rapidly respond to selection. First, there is enough dormant genetic variation with respect to brain size for selection to respond to, as demonstrated by effects of domestication on brain size in numerous endotherms (Balcarcel et al. 2022) and many recent selection experiments in fishes (e.g., Kotrschal et al. 2013). Second, brains are among the energetically most expensive organs in the body per unit weight, yet brain size varies by orders of magnitude among vertebrate species (Jerison 1973; Tsuboi et al. 2018). Finally, relative brain size increased over evolutionary time in many birds and mammals (Balanoff et al. 2013; Jerison 1973; Rowe et al. 2011), including primates (Gilbert and Jungers 2017; Melchionna et al. 2019; Smaers et al. 2021). Nonetheless, it is important to test the assumption of adaptive variation, especially because some scholars have argued that brain size is not an adaptive trait (e.g., Martin 1996) or that there may be a long evolutionary lag between changes in brain size when body size changes (e.g., Aboitiz 1996; Grabowski 2016).

Larger‐brained animals are known to have slower development, because brain growth limits the development process and more encephalized species therefore take longer to mature (Halley and Deacon 2017; Sacher and Staffeldt 1974; Workman et al. 2013). This inevitably results in a later onset of reproduction in larger‐brained species (Barton and Capellini 2011; Schuppli et al. 2012). The effect on reproductive rates also tends to be negative (Isler and van Schaik 2009; Todorov et al. 2021), as it reflects the balance of the costs of producing larger newborns (Isler and van Schaik 2009; Song et al. 2025) and the possible benefit of more efficient foraging. On the other hand, a benefit of a larger brain is longer lifespan, which is the selective response to higher adult survival (Stearns 1992). Coevolution between brain size and maximum lifespan is found for both mammals (González‐Lagos et al. 2010) and birds (Jiménez‐Ortega et al. 2020), but this trend is not universal in these lineages (DeCasien et al. 2018; Powell et al. 2019). Taken together, these changes in survival and reproduction are likely to reduce a population's net reproductive rate, R_0_, with an increase in brain size, and should thus be selected against unless larger brains have some hitherto unrecorded countervailing advantage.

To estimate the marginal effects of brain size changes on the three major parameters (development time, adult survival, and adult reproductive rate) within a given population, and thus their combined effect on *R*_0_ would ideally require selection experiments in nature. Because this is obviously unrealistic, we must rely on a crude estimate based on interspecific trends in precocial, monotokous mammals, including primates (Isler and van Schaik 2009). This exercise suggests that the result will be reduced population viability to the point of *R*_0_ < 1, and thus non‐viability. Comparative analyses across primate species show that for a 1 standard deviation (SD) increase in standardized relative brain size (i.e., controlling for the effect of body mass), standardized age at first reproduction is delayed by 0.6 SD and standardized annual birth rate is reduced by 0.5 SD, whereas standardized maximum lifespan is increased by a mere 0.2 SD (Isler and van Schaik 2009). This modest lengthening of adult lifespan is likely not nearly big enough to compensate for the dual cost of delay in reproduction and lower reproductive rate. We must be cautious in applying estimates based on interspecific patterns because they need not translate directly to within‐population effect sizes. However, the same conclusion emerges from another interspecific approach: *r*_max_, the population growth under ideal conditions of zero mortality. In the largest‐brained primate species, *r*_max_ is already dangerously close to zero (primates: Isler and van Schaik 2012). Very large‐brained species therefore flirt with demographic non‐viability and thus population extinction (Chevin et al. 2010; van Schaik 2013), unless they can reduce mortality to the absolute minimum.

Here, we suggest that we have so far overlooked another important positive fitness effect of increased brain size, namely increased immature survival. Comparative studies have revealed that brain size evolution was greatly facilitated by the emergence of extended parental provisioning (Griesser et al. 2023; Song et al. 2025), because without parental provisioning young could not sustain the growth of large brains. Across endothermic vertebrates, parental provisioning coevolved with the evolution of larger brains (Song et al. 2025; van Schaik et al. 2023). However, selection on larger offspring at birth in more encephalized lineages is favored only when survival of young is increased (Anderson and Gillooly 2021). Given that larger‐brained species do have larger young at birth and at independence, the offspring of larger‐brained species may well survive better than those of smaller‐brained species. We therefore hypothesize that this improvement in immature survival could make a major contribution to making larger brains adaptive.

There are currently no published comparative analyses of immature survival in relation to brain size in any lineage. Here, we provide an analysis based on all available studies on pre‐reproductive survival in primates that report the relevant information from natural, non‐provisioned populations. Although the sample is small (18 species), the species vary greatly in brain and body size. We examined survival until the age at first reproduction (AFR) in females (S_A_).

**Figure 1:**
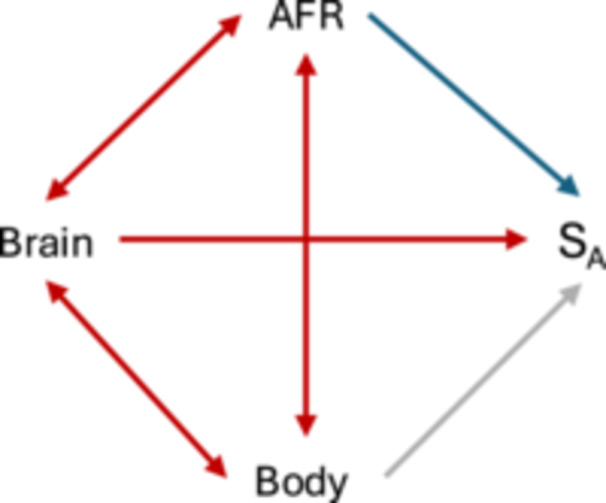
The predicted direct effects of brain size, body size and age at first reproduction (AFR) on the survival until AFR (S_A_), and the potentially confounding effects of the correlations among the three independent variables, which may affect the strength or even sign of these direct effects.

We predict the following pattern (Figure 1). When it takes longer to reach AFR, this should, other things being equal, negatively affect S_A_, whereas brain mass should positively affect S_A_. The effect of body mass is unspecified: larger animals take longer to mature, which should cause a negative effect, but larger size may also reduce vulnerability to predation, which has a positive effect (Figure 1).

The main challenge is the high collinearity among the three independent variables. We alleviated this problem in three different ways. First, we took either residual brain size or absolute brain size as the independent variable in phylogenetic regression models and compared the results. Second, we also examined which of the possible independent variables most directly affect S_A_ by conducting a phylogenetic path analysis. Finally, we examined survival during the first year (S_1_) and so removed the effect of AFR, which allowed us to distinguish between the potentially negative effect of AFR and the positive one of its correlate, body mass.

## 2 Methods

### 2.1 Data on Animals

We took data from all available (to the best of our knowledge) published long‐term studies of wild primate populations (non‐provisioned, non‐hunted, and living in largely undisturbed natural habitats, with natural predators). By focusing on populations living in the conditions in which these species evolved and continue to be exposed to selection, we indirectly control for many of the more immediate variables affecting survival such as predation, climatic events, or and starvation. Supporting Information S1: Table S1 lists the data and their sources.

Estimates of immature survival and age at first reproduction (AFR) were almost always from the same population. We took these measures for females only, so as to control for variation in male mortality and AFR linked to the mating system. All weight data from brain and body mass are also for females. Brain data were mostly taken from (Heldstab et al. 2018; Isler et al. 2008). Although the samples were mostly not from the same population, we do not expect this to be add substantial error owing to the large range of values across species.

Size and age variables were log‐transformed, and survival rates were logit‐transformed to generate normal distributions. All variables were standardized (Z‐transformed) before analysis, such that each variable had a mean of zero and a standard deviation of one.

### 2.2 Statistical Methods

All statistical analyses were carried out in the R 4.1.1 environment (Team 2013). We used phylogenetic generalized least squares (PGLS) regression models in the R package *phylolm* (Tung Ho and Ané 2014) to test the effects of brain mass on S_A_ while controlling for the potentially confounding effects of body mass and AFR. To avoid spurious effects owing to the high collinearity between these three variables, we took various approaches. First, we considered absolute brain mass as the independent variable, either alone or with the two others. Second, although this approach is debated (Freckleton 2009; Rogell et al. 2020), we also used residual brain mass (from the log‐log regression of brain on body mass) as an independent variable in the same combinations to see if this approach removed collinearity problems. We then applied model selection to identify the best‐supported representation of the causal relationships among the various combinations of AFR, body mass, or residual brain mass in their effect on S_A_. Third, we examined S_1_ to remove the effect of AFR, and so reveal a possible independent effect of body size, otherwise masked by collinearity.

To control for phylogenetic nonindependence, we used a recently published time‐calibrated multi‐tree phylogeny of primates (Wisniewski et al. 2022), and randomly selected 100 trees to generate a maximum clade credibility (MCC) tree.

Finally, to check the robustness of the PGLS results, we also directly tested the proposed causal structure of Figure 1 by conducting a phylogenetic path analysis (PPA). The candidate PPA models were constructed by considering whether brain size, body size, or AFR had a direct effect on survival to AFR, while also accounting for potential correlations among these variables. For both absolute and residual brain size, we tested 42 candidate models, (see Supporting Information S1: Figure S1 for details), using the *phylopath* (van der Bijl 2018) package in R. For each model, we assessed the implied conditional independencies (i.e., d‐separation statements) using Fisher's *C*‐statistic. Models that passed the *C* test (*p* > 0.05) were considered consistent with the data and were ranked using the CICc (*C*‐statistic Information Criterion corrected for small sample size).

## 3 Results

**Figure 2:**
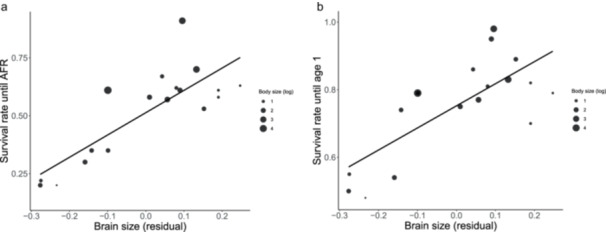
The statistical effect of residual female brain size on female survival until age at first reproduction (a) or female survival until age 1 (b) across primate species. The size of the symbols reflects the (log) body size of the species concerned.

The model selection to identify the best‐fitting combination of age at first reproduction (AFR), body mass, absolute brain mass, or residual brain mass in their effect on S_A_ yielded two best models of equal fit (Table 1). These included body mass and brain mass, the latter as either residual or absolute size (Table 1). We confirmed that the effects on S_A_ of both the absolute (Table 2a) and residual brain mass (Table 2b) were both significant, but that the significant effect of body mass changed sign. However, the model with absolute brain mass is not reliable, because the collinearity of the variables remained prohibitively high (Model 3 in Table 3). In contrast, the model with residual brain mass has negligible collinearity (Model 4 in Table 3) and is thus more reliable. It suggests independent positive effects of both brain mass and body mass on S_A_. Overall, S_A_ appears to be affected more strongly by brain mass than by body mass (Figure 2 and Table 1).

**Table 1:** Candidate models to test the effect of body mass (log), age of first reproduction (AFR) and both absolute log brain and residual brain mass on S_A_ (survival rate until AFR). An x indicates that a variable was not included in the particular model. Best‐fitting models with lowest AIC values are indicated in bold, and the significance of the effect of each variables is indicated by *p* < 0.05 *, *p* < 0.01 **, and *p* < 0.001 ***.

Model structure	AFR (log)	Body mass (log)	Brain mass (residual)	Brain mass (log)	df	logLik	AICc
Br_Body	x	−1.023***	x	1.680***	5	−9.784	**34.568**
resBr_Body	x	0.468***	0.774***	x	5	−9.784	**34.568**
resBr_AFR	0.507**	x	0.489**	x	5	−11.596	38.193
resBr_All	0.170	0.357	0.678**	x	6	−9.401	38.439
Br_All	0.170	−0.951**	x	1.473**	6	−9.401	38.439
AFR	0.575**	x	x	x	4	−13.849	38.774
resBr	x	x	0.595**	x	4	−13.906	38.889
Br_AFR	0.498*	x	x	0.162	5	−13.618	42.236
AFR_Body	0.592**	−0.056	x	x	5	−13.797	42.593
NULL	x	x	x	x	3	−18.173	44.060
Br	x	x	x	0.769***	4	−16.885	44.847
Body	x	0.110	x	x	4	−18.039	47.155

**Table 2:** Effect of brain mass (log and residual) and body mass on a) S_A_ (survival rate until female AFR), and b) S_1_ (survival rate until age 1).

	Estimate	se	*t*	*p*
(a) Survival rate until AFR (S_A_) with absolute brain size				
Intercept	0.000	0.111	−0.001	0.999
Brain mass (log)	1.680	0.242	6.950	< 0.001***
Body mass (log)	−1.023	0.241	−4.245	< 0.001***
(b) Survival rate until AFR (S_A_) with relative brain size				
Intercept	0.000	0.111	−0.001	0.999
Brain mass (residual)	0.774	0.111	6.950	< 0.001***
Body mass (log)	0.468	0.111	4.215	< 0.001***
(c) Survival rate until age 1 (S_1_) with absolute brain size				
Intercept	0.000	0.173	0.001	1.000
Brain mass (log)	1.334	0.378	3.533	0.003**
Body mass (log)	−0.763	0.377	−2.027	0.061
(d) Survival rate until age 1 (S_1_) with relative brain size				
Intercept	0.000	0.173	0.001	1.000
Brain mass (residual)	0.614	0.174	3.533	0.003**
Body mass (log)	0.421	0.173	2.428	0.028*

**Table 3:** The variance inflation factors of the models testing the effect of brain mass (log and residual), body mass, and AFR on S_A_.

Variables	Model 1	Model 2	Model 3	Model 4
AFR	3.763	3.763		
Body mass (log)	5.408	2.602	4.720	1.000
Brain mass (log)	10.262		4.720	
Brain mass (residual)		2.174		1.000

AFR did not enter the two best‐fitting models. It played a role only when we removed body mass from the model. We can also directly exclude the role of AFR by focusing on survival rate until age one (S_1_). Table 2c and d shows the models with absolute and relative brain size as independent variables. The pattern is similar as for S_A_. Brain size, residual or absolute, is the best predictor of S_1_. Thus, the positive brain mass effect on immature survival is already apparent at age 1 (as illustrated in Figure 2), but the higher effect sizes for S_A_ suggest that the brain size effect continues beyond the first year. However, in the model with residual brain mass, there was a weaker, but significant positive effect of body size as well (Table 2b).

**Figure 3:**
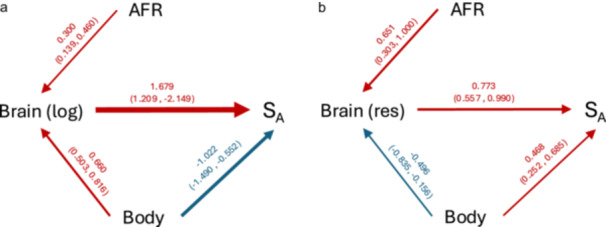
Structure of the relationships in the most parsimonious path models (see Supporting Information S1: Table S2), based on (a) log‐transformed brain size and (b) residual brain size. Abbreviations: AFR = age at first reproduction; S_A_ = survival until AFR. Path coefficients are shown as means with 95% confidence intervals in parentheses. Arrow thickness indicates the level of statistical support, and arrow color denotes the direction of the relationship (red = positive; blue = negative).

To check these conclusions, we also conducted a phylogenetic path analysis (Supporting Information S1: Figure S1). A single best‐supported model was identified (ΔCICc < 2, Supporting Information S1: Table S2) and path coefficients are shown in Figure 3. It shows that S_A_ is affected by brain size and body size but not by AFR, as found before, regardless of whether we include brain size (Figure 3a) or residual brain size (Figure 3b).

## 4 Discussion

Larger brain size is associated with a later age at first reproduction (AFR). Thus, if brain size does not affect mortality risk during immaturity, larger‐brained species should have lower S_A_. Yet, we find no negative effect of AFR and instead a positive effect of brain size on immature survival (regardless of using absolute or residual brain size). In the most reliable model, adult body mass also shows a positive effect, which probably reflects the lower vulnerability to predation of larger‐bodied species in general, but the effect of adult brain size is about twice as strong. This effect is already visible at S_1_, which confirms the lack of effect of time of exposure to preadult mortality (AFR) and is consistent with the effects of both brain and body size.

These results help to solve the problem of whether larger brains are adaptive, and if so, why exactly. Larger brains improve survival, not just of adults, but also, and perhaps especially, of immatures. Thus, although the reduction of *r*_max_ relative to the state with smaller brains remains strong, a reduction of mean *R*_0_ is no longer inevitable. Moreover, an additional major advantage may obtain. Larger‐brained taxa are known to have greater population stability (primates: Morris et al. 2011; birds: Fristoe et al. 2017), and thus reduced risk of population extinction. Comparative analyses cannot determine the relative importance of the immediate effect on *R*_0_ vs. the longer‐term effect on population stability. To evaluate this, we need careful demographic modelling of realistic marginal effects of changes in brain size on each fitness component, and so on overall fitness, relative to the ancestral state.

The mechanism underlying the brain size effect is presumably parental protection and provisioning. The mere association with young, linked to postnatal provisioning and high parental activity levels, provides a safe haven (Griesser et al. 2006), likely to keep them away from various threats. In addition, parents or other caretakers can be vigilant, give alarm calls and even mob potential predators, all of which improve juvenile survival (Fichtel 2012). Finally, provisioning allows for relatively risk‐free maintenance during a phase of high rate of brain growth, which allows the infants to avoid high levels of risky supplementary foraging (Janson and van Schaik 1993), and buys them time to learn vital skills, partly through social learning, for adult life (Schuppli et al. 2012). Adults of larger‐brained species are generally more effective in all these activities, as shown by the correlation with adult survival (mammals: González‐Lagos et al. 2010; birds: Jiménez‐Ortega et al. 2020), although examining the exceptions (cf. DeCasien et al. 2018) may be instructive. These results are therefore consistent with the recent finding that the lineages in which brain size has shown evolutionary increases over time (birds and mammals: Balanoff et al. 2013; Rowe et al. 2011) are those in which both pre‐ and postnatal parental provisioning has burgeoned (Song et al. 2025).

Primates are among the vertebrate orders with the largest relative brain size (Smaers et al. 2021), and it is possible that the positive impact of brain size on survival is evident only above a particular size, so that less encephalized taxa may not show this effect. Various other factors could also be at play. For instance, among carnivores with extensive allomaternal care, larger brains are associated with shorter adult lifespans (Isler and van Schaik 2009), but that same care may raise reproductive rates. The adult survival effect of large brains is also absent in cetaceans, potentially related to various risk factors linked to life as a marine mammal (DeCasien et al. 2018). To what extent these effects also obtain for immature survival remains unexamined. Thus, a broader study of immature mortality across all mammals and birds may reveal to what extent brain size affects immature mortality and also suggest which parental actions are most effective in increasing their offspring's immature survival.

An alternative explanation for the improved survival of immatures is that it is a byproduct of the slowdown of life history (Zipple et al. 2024), rather than caused by increased adult brain size and its consequences. Because dependent young show strongly reduced survival when they lose their mother (primates: van Noordwijk 2012), females obviously improve their fitness by staying alive while they have dependent offspring. For selection to favor this, females must improve their own survival and therefore reduce reproductive rate, which is competing for energetic investment with survival. Zipple et al. (2024) modeled various variants of this effect, and showed that both immature survival and female lifespan are increased by this. This mechanism may thus provide an alternative explanation for the correlation between S_A_ and adult brain size, because of the well‐known coevolution between brain size and longevity (see above). On the other hand, it is also possible that this outcome may actually be at least partially achieved through increased brain size, which is not included in the Zipple model.

To test between these alternatives, we can ask whether adult survival is a better predictor of immature survival than adult brain size. In a preliminary test we used maximum adult lifespan of the species involved,that is, longevity—AFR (with longevity often obtained from captive animals; see Supporting Information S1: Table S3 for sources). We found that residual brain size remains a significant predictor of both S_A_ and S_1_, whereas maximum adult lifespan is not (Supporting Information S1: Table S3). Thus, the brain size effect is stable, and may in fact explain part of the maternal survival effect examined in the Zipple et al. (2024) model. However, for a more comprehensive test it would be good to use life expectancy at AFR from full life tables of wild populations (a direct measure of maternal survival) and a broader sample of mammals (and birds), so as to reduce the strong collinearity between adult brain size and life history variables (see above).

Looking beyond endotherms, it is possible that the effect on immature survival revealed here for primates does not hold in most ectothermic vertebrates, for whom the brain size effect on adult mortality may be weak or even negative among them (Fischer and Jungwirth 2022; Stark and Pincheira‐Donoso 2022; but see Yu et al. 2018). First, in many of them, there is no association between parents and offspring (Balshine 2012), and the protection component is therefore absent. Second, in many others, even where there is post‐hatching association, there is no parental provisioning in the great majority of species (Balshine 2012). It is thus possible that among many ectotherms opportunities for parents to improve the survival of their offspring are limited, and that this acted as an evolutionary constraint on brain size enlargement (encephalization). These limitations on offspring protection and provisioning in many ectotherms may help to explain why some ectotherms with relatively large hatchlings nonetheless have small brains (Song et al. 2025). Alternatively, the advantage of larger relative brain size may be linked to adult reproduction rather than survival: at least among fishes, larger adults produce disproportionately larger offspring and larger clutches (Barneche et al. 2018). The different life‐history effects of brain size between ectotherms and endotherms therefore deserve closer study.

## Author Contributions

Zitan Song: conception, analyses and writing. Carel P. van Schaik: conception, data preparation, and writing.

## Ethics Statement

The authors have nothing to report.

## Conflicts of Interest

The authors declare no conflicts of interest.

## Supporting information


**Figure S1:** 42 candidate path models were tested using phylogenetic path analysis to explore the relationships among body size, brain size, age at first reproduction (AFR), and survival until AFR (Surv_AFR). Models were evaluated based on d‐separation tests, with overall fit quantified using Fisher's C statistic and ranked using CICc. **Table S1:** Data on body mass, brain mass, age at first reproduction and immature survival of female primates in 18 species living in natural, non‐provisioned populations. **Table S2:** Model selection results from phylogenetic path analysis. Each candidate model was evaluated using Fisher's C test and ranked by the C‐statistic Information Criterion corrected for small sample size (CICc). Model structures are shown in Figure S1. **Table S3:** Effect of brain mass (residual) and max adult life span (max life span ‐ AFR) on **a**) S_A_ (survival rate until female AFR), and **b**) S_1_ (survival rate until age 1).

## Data Availability

The data is provided in the Supporting Information S1.
