# Supplementary material for: Slowly but Surely: Larger Brains Improve Immature Survival in Primates
Source: Am J Primatol. 2025 Aug 29;87(9):e70072. doi: 10.1002/ajp.70072 (PMC12397558; doi:10.1002/ajp.70072)
Supplement: Supplementary file 1 — Figure S1: 42 candidate path models were tested using phylogenetic path analysis to explore the relationships among body size, brain size, age at first reproduction (AFR), and survival until AFR (Surv_AFR). Models were evaluated based on d‐separation tests, with overall fit quantified using Fisher's C statistic and ranked using CICc. Table S1: Data on body mass, brain mass, age at first reproduction and immature survival of female primates in 18 species living in natural, non‐provisioned populations. Table S2: Model selection results from phylogenetic path analysis. Each candidate model was evaluated using Fisher's C test and ranked by the C‐statistic Information Criterion corrected for small sample size (CICc). Model structures are shown in Figure S1. Table S3: Effect of brain mass (residual) and max adult life span (max life span ‐ AFR) on a) SA (survival rate until female AFR), and b) S1 (survival rate until age 1). [file AJP-87-e70072-s001.docx]

**Supplementary materials**

**Slowly but surely: Larger brains improve immature survival in primates**

Zitan Song^1*^ & Carel P. van Schaik^1, 2,^ ^3^

**Table S1.**

Data on body mass, brain mass, age at first reproduction and immature survival of female primates in 18 species living in natural, non-provisioned populations.

| \| **Genus** \| **Species** \| **Bo ADF (kg)** \| **Br ADF (g)** \| **AFR (yr)** \| **Surv -AFR** \| **Surv-1st yr** \| **Source (references and footnotes)** \| \| --- \| --- \| --- \| --- \| --- \| --- \| --- \| --- \| \| *Propithecus* \| *diadema edwardsii* \| 5.5 \| 38.3 \| 5.5 \| 0.2 \| 0.5 \| Ranomafana from Pochron et al. 2004 \| \| *Propithecus* \| *verreauxi* \| 3.2 \| 26.2 \| 5.0 \| 0.22 \| 0.55 \| Kirindy from Kappeler & Fichtel 2012 (1) \| \| *Lemur* \| *catta* \| 2.2 \| 22.1 \| 3.23 \| 0.20 \| 0.48 \| Beza from Gould et al. 2003 (2) \| \| *Alouatta* \| *palliata* \| 5.35 \| 51.2 \| 4.0 \| 0.35 \| 0.74 \| BCI from Fröhlich et al. 1981 \| \| *Alouatta* \| *seniculus* \| 5.2 \| 55.4 \| 5.1 \| 0.35 \| 0.79 \| Hato Masaguaral from Crockett, in Table in Robinson 1988 \| \| *Cebus* \| *capucinus* \| 2.3 \| 69 \| 6.5 \| 0.63 \| 0.79 \| Lomas from Bronikowski et al. 2016 \| \| *Cebus* \| *nigritus* \| 2.5 \| 64.2 \| 6.4 \| 0.58 \| 0.70 \| Iguazu from Janson et al. 2012; AFR from Di Bitetti & Janson 2001 (3) \| \| *Cebus* \| *olivaceus* \| 2.5 \| 64.2 \| 7.0 \| 0.61 \| 0.82 \| Hato Masaguaral from Robinson 1988 (4). \| \| *Brachyteles* \| *arachnoides* \| 8.33 \| 119.4 \| 8.5 \| 0.61 \| 0.95 \| Montes Claros from Bronikowski et al. (2016); AFR from Martins & Strier 2004; brain from Burger et al. 2019 \| \| *Presbytis* \| *thomasi* \| 6.7 \| 57.7 \| 5.4 \| 0.3 \| 0.54 \| Ketambe from Wich et al. 2007 (5) \| \| *Cercopithecus* \| *mitis* \| 4.2 \| 66 \| 7.5 \| 0.67 \| 0.86 \| Kakamega from Bronikowski et al. 2016 \| \| *Papio* \| *cynocephalus* \| 12.8 \| 150 \| 5.5 \| 0.57 \| 0.77 \| Amboseli from Bronikowski et al. 2016 \| \| *Macaca* \| *fascicularis* \| 3.5 \| 63.2 \| 5.2 \| 0.62 \| 0.81 \| Ketambe from van Noordwijk & van Schaik 1999 \| \| *Macaca* \| *fuscata* \| 8.03 \| 96.8 \| 6.1 \| 0.58 \| 0.75 \| Yakushima, from Takahata et al. 1998 \| \| *Hylobates* \| *lar* \| 5.4 \| 101.5 \| 10.5 \| 0.53 \| 0.89 \| Reichard & Barelli 2008; Reichard et al. 2012. (6) \| \| *Gorilla* \| *beringei* \| 95 \| 433 \| 9.5 \| 0.61 \| 0.79 \| Karisoke from Bronikowski et al. 2016 \| \| *Pongo* \| *pygmaeus* \| 35.5 \| 338 \| 14.8 \| 0.91 \| 0.98 \| van Noordwijk et al. 2018 \| \| *Pan* \| *troglodytes* \| 34 \| 357 \| 14.0 \| 0.7 \| 0.83 \| Wood et al. 2017; AFR from Robson et al. 2006 (7) \| |
| --- | --- | --- | --- | --- | --- | --- | --- | --- | --- | --- | --- | --- | --- | --- | --- | --- | --- | --- | --- | --- | --- | --- | --- | --- | --- | --- | --- | --- | --- | --- | --- | --- | --- | --- | --- | --- | --- | --- | --- | --- | --- | --- | --- | --- | --- | --- | --- | --- | --- | --- | --- | --- | --- | --- | --- | --- | --- | --- | --- | --- | --- | --- | --- | --- | --- | --- | --- | --- | --- | --- | --- | --- | --- | --- | --- | --- | --- | --- | --- | --- | --- | --- | --- | --- | --- | --- | --- | --- | --- | --- | --- | --- | --- | --- | --- | --- | --- | --- | --- | --- | --- | --- | --- | --- | --- | --- | --- | --- | --- | --- | --- | --- | --- | --- | --- | --- | --- | --- | --- | --- | --- | --- | --- | --- | --- | --- | --- | --- | --- | --- | --- | --- | --- | --- | --- | --- | --- | --- | --- | --- | --- | --- | --- | --- | --- | --- | --- | --- | --- | --- | --- | --- |
| Bo ADF = adult female body mass; Br ADF = adult female brain mass; AFR = age at first reproduction |
| **Footnotes** |
| (1) Brain size taken from species mean value. We selected Kirindy rather than Beza because it has natural predators. |
| (2) Survival to AFR is a rather crude estimate. |
| (3) Body and brain from *Cebus apella* (closest relative, since robust, tufted capuchin). |
| (4) Brain mass from *Cebus (Sapajus) apella*, which is closest in weight. |
| (5) We took brain size from *P. melalophos*- its geographic sister species. |
| (6) Survival to AFR is average between 0.46 reported by Reichard & Barelli 2008 and 0.60 reconstructed from Reichard et al. 2012. |
| (7) We took Ngogo because it is least affected by (human-introduced) disease and has no declining population. |
|  |
| **References** |
| Pochron, S. T., Tucker, W. T., & Wright, P. C. (2004). Demography, life history, and social structure in *Propithecus diadema* edwardsi from 1986–2000 in Ranomafana National Park, Madagascar. *American Journal of Physical Anthropology: The Official Publication of the American Association of Physical Anthropologists*, *125*(1), 61-72. |
| Kappeler, P. M., & Fichtel, C. (2012). A 15-year perspective on the social organization and life history of sifaka in Kirindy Forest. *Long-term field studies of primates*, 101-121. |
| Gould, L., Sussman, R. W., & Sauther, M. L. (2003). Demographic and life‐history patterns in a population of ring‐tailed lemurs (*Lemur catta*) at Beza Mahafaly Reserve, Madagascar: a 15‐year perspective. *American Journal of Physical Anthropology: The Official Publication of the American Association of Physical Anthropologists*, *120*(2), 182-194. |
| Froehlich, J. W., Thorington, R. W., & Otis, J. S. (1981). The demography of howler monkeys (*Alouatta palliata*) on Barro Colorado Island, Panama. *International Journal of Primatology*, *2*, 207-236. |
| Robinson, J. G. (1988). Demography and group structure in wedge-capped capuchin monkeys, *Cebus olivaceus*. *Behaviour*, *104*(3-4), 202-232. |
| Bronikowski, A. M., Cords, M., Alberts, S. C., Altmann, J., Brockman, D. K., Fedigan, L. M., ... & Morris, W. F. (2016). Female and male life tables for seven wild primate species. *Scientific data*, *3*(1), 1-8. |
| Janson, C., Baldovino, M. C., & Di Bitetti, M. (2012). The group life cycle and demography of brown capuchin monkeys (*Cebus apella nigritus*) in Iguazú National Park, Argentina. *Long-term field studies of primates*, 185-212. |
| Di Bitetti, M. S., & Janson, C. H. (2001). Reproductive socioecology of tufted capuchins (*Cebus apella nigritus*) in northeastern Argentina. *International Journal of Primatology*, *22*, 127-142. |
| Martins, W. P., & Strier, K. B. (2004). Age at first reproduction in philopatric female muriquis (*Brachyteles arachnoides* *hypoxanthus*). *Primates*, *45*, 63-67. |
| Burger, J. R., George Jr, M. A., Leadbetter, C., & Shaikh, F. (2019). The allometry of brain size in mammals. *Journal of Mammalogy*, *100*(2), 276-283. |
| Wich, S. A., Steenbeek, R., Sterck, E. H., Korstjens, A. H., Willems, E. P., & Van Schaik, C. P. (2007). Demography and life history of Thomas langurs (*Presbytis thomasi*). *American Journal of Primatology: Official Journal of the American Society of Primatologists*, *69*(6), 641-651. |
| van Noordwijk, M. A., & van Schaik, C. P. (1999). The effects of dominance rank and group size on female lifetime reproductive success in wild long-tailed macaques, *Macaca fascicularis*. *Primates*, *40*, 105-130. |
| Takahata, Y., Suzuki, S., Agetsuma, N., Okayasu, N., Sugiura, H., Takahashi, H., ... & Sprague, D. S. (1998). Reproduction of wild Japanese macaque females of Yakushima and Kinkazan Islands: a preliminary report. *Primates*, *39*, 339-349. |
| Reichard, U. H., & Barelli, C. (2008). Life history and reproductive strategies of Khao Yai Hylobates lar: implications for social evolution in apes. *International Journal of Primatology*, *29*, 823-844. |
| Reichard, U. H., Ganpanakngan, M., & Barelli, C. (2012). White-handed gibbons of Khao Yai: social flexibility, complex reproductive strategies, and a slow life history. *Long-term field studies of primates*, 237-258. |
| van Noordwijk, M. A., Atmoko, S. S. U., Knott, C. D., Kuze, N., Morrogh-Bernard, H. C., Oram, F., ... & Willems, E. P. (2018). The slow ape: High infant survival and long interbirth intervals in wild orangutans. *Journal of Human Evolution*, *125*, 38-49. |
| Wood, B. M., Watts, D. P., Mitani, J. C., & Langergraber, K. E. (2017). Favorable ecological circumstances promote life expectancy in chimpanzees similar to that of human hunter-gatherers. *Journal of Human Evolution*, *105*, 41-56. |
| Robson, S. L., Van Schaik, C. P., & Hawkes, K. (2006). The derived features of human life history. *The evolution of human life history*, Eds Hawkes, K & Paine, RR, *17-44,* School of American Research Press. |

**Table S2.**

Model selection results from phylogenetic path analysis. Each candidate model was evaluated using Fisher’s C test and ranked by the C-statistic Information Criterion corrected for small sample size (CICc). Model structures are shown in Figure S1.

| Models | k | q | Fisher’s C | p | CICc | Delta CICc | weight |
| --- | --- | --- | --- | --- | --- | --- | --- |
| **Brain (log)** | | | | | | | |
| **m5_4** | **2** | **8** | **4.411** | **0.353** | **36.411** | **0.000** | **0.712** |
| m3_4 | 2 | 8 | 8.867 | 0.065 | 40.867 | 4.455 | 0.077 |
| m1_4 | 1 | 9 | 1.594 | 0.451 | 42.094 | 5.682 | 0.042 |
| m4_4 | 1 | 9 | 1.594 | 0.451 | 42.094 | 5.682 | 0.042 |
| m6_4 | 1 | 9 | 1.594 | 0.451 | 42.094 | 5.682 | 0.042 |
| m5_3 | 3 | 7 | 17.052 | 0.009 | 42.252 | 5.841 | 0.038 |
| m2_4 | 2 | 8 | 13.739 | 0.008 | 45.739 | 9.327 | 0.007 |
| m7_4 | 2 | 8 | 13.739 | 0.008 | 45.739 | 9.327 | 0.007 |
| m1_3 | 2 | 8 | 14.235 | 0.007 | 46.235 | 9.823 | 0.005 |
| m4_3 | 2 | 8 | 14.235 | 0.007 | 46.235 | 9.823 | 0.005 |
| m6_3 | 2 | 8 | 14.235 | 0.007 | 46.235 | 9.823 | 0.005 |
| m3_3 | 3 | 7 | 21.507 | 0.001 | 46.707 | 10.296 | 0.004 |
| m5_5 | 2 | 8 | 14.752 | 0.005 | 46.752 | 10.341 | 0.004 |
| m1_1 | 2 | 8 | 16.183 | 0.003 | 48.183 | 11.771 | 0.002 |
| m4_1 | 2 | 8 | 16.183 | 0.003 | 48.183 | 11.771 | 0.002 |
| m5_6 | 2 | 8 | 16.536 | 0.002 | 48.536 | 12.124 | 0.002 |
| m5_1 | 3 | 7 | 23.759 | 0.001 | 48.959 | 12.548 | 0.001 |
| m6_1 | 2 | 8 | 18.288 | 0.001 | 50.288 | 13.877 | 0.001 |
| m5_2 | 3 | 7 | 25.985 | 0.000 | 51.185 | 14.774 | 0.000 |
| m3_5 | 2 | 8 | 19.207 | 0.001 | 51.207 | 14.796 | 0.000 |
| m2_3 | 3 | 7 | 26.379 | 0.000 | 51.579 | 15.168 | 0.000 |
| m7_3 | 3 | 7 | 26.379 | 0.000 | 51.579 | 15.168 | 0.000 |
| m1_5 | 1 | 9 | 11.935 | 0.003 | 52.435 | 16.023 | 0.000 |
| m4_5 | 1 | 9 | 11.935 | 0.003 | 52.435 | 16.023 | 0.000 |
| m6_5 | 1 | 9 | 11.935 | 0.003 | 52.435 | 16.023 | 0.000 |
| m3_6 | 2 | 8 | 20.991 | 0.000 | 52.991 | 16.580 | 0.000 |
| m3_1 | 3 | 7 | 28.215 | 0.000 | 53.415 | 17.003 | 0.000 |
| m2_1 | 3 | 7 | 28.327 | 0.000 | 53.527 | 17.116 | 0.000 |
| m1_6 | 1 | 9 | 13.718 | 0.001 | 54.218 | 17.807 | 0.000 |
| m4_6 | 1 | 9 | 13.718 | 0.001 | 54.218 | 17.807 | 0.000 |
| m6_6 | 1 | 9 | 13.718 | 0.001 | 54.218 | 17.807 | 0.000 |
| m4_2 | 2 | 8 | 23.168 | 0.000 | 55.168 | 18.756 | 0.000 |
| m6_2 | 2 | 8 | 23.168 | 0.000 | 55.168 | 18.756 | 0.000 |
| m7_1 | 3 | 7 | 30.433 | 0.000 | 55.633 | 19.221 | 0.000 |
| m2_5 | 2 | 8 | 24.080 | 0.000 | 56.080 | 19.668 | 0.000 |
| m7_5 | 2 | 8 | 24.080 | 0.000 | 56.080 | 19.668 | 0.000 |
| m2_6 | 2 | 8 | 25.863 | 0.000 | 57.863 | 21.452 | 0.000 |
| m7_6 | 2 | 8 | 25.863 | 0.000 | 57.863 | 21.452 | 0.000 |
| m1_2 | 2 | 8 | 26.332 | 0.000 | 58.332 | 21.921 | 0.000 |
| m3_2 | 3 | 7 | 33.605 | 0.000 | 58.805 | 22.394 | 0.000 |
| m2_2 | 3 | 7 | 46.333 | 0.000 | 71.533 | 35.121 | 0.000 |
| m7_2 | 3 | 7 | 46.333 | 0.000 | 71.533 | 35.121 | 0.000 |
| **Brain (residual)** | | | | | | | |
| **m5_4** | **2** | **8** | **4.411** | **0.353** | **36.411** | **0.000** | **0.665** |
| m5_5 | 2 | 8 | 8.201 | 0.084 | 40.201 | 3.789 | 0.100 |
| m1_4 | 1 | 9 | 1.594 | 0.451 | 42.094 | 5.682 | 0.039 |
| m4_4 | 1 | 9 | 1.594 | 0.451 | 42.094 | 5.682 | 0.039 |
| m6_4 | 1 | 9 | 1.594 | 0.451 | 42.094 | 5.682 | 0.039 |
| m5_3 | 3 | 7 | 17.052 | 0.009 | 42.252 | 5.841 | 0.036 |
| m3_4 | 2 | 8 | 11.126 | 0.025 | 43.126 | 6.714 | 0.023 |
| m2_4 | 2 | 8 | 13.739 | 0.008 | 45.739 | 9.327 | 0.006 |
| m7_4 | 2 | 8 | 13.739 | 0.008 | 45.739 | 9.327 | 0.006 |
| m1_5 | 1 | 9 | 5.383 | 0.068 | 45.883 | 9.472 | 0.006 |
| m4_5 | 1 | 9 | 5.383 | 0.068 | 45.883 | 9.472 | 0.006 |
| m6_5 | 1 | 9 | 5.383 | 0.068 | 45.883 | 9.472 | 0.006 |
| m1_3 | 2 | 8 | 14.235 | 0.007 | 46.235 | 9.823 | 0.005 |
| m4_3 | 2 | 8 | 14.235 | 0.007 | 46.235 | 9.823 | 0.005 |
| m6_3 | 2 | 8 | 14.235 | 0.007 | 46.235 | 9.823 | 0.005 |
| m3_5 | 2 | 8 | 14.915 | 0.005 | 46.915 | 10.504 | 0.003 |
| m1_1 | 2 | 8 | 16.101 | 0.003 | 48.101 | 11.690 | 0.002 |
| m4_1 | 2 | 8 | 16.101 | 0.003 | 48.101 | 11.690 | 0.002 |
| m5_6 | 2 | 8 | 16.536 | 0.002 | 48.536 | 12.124 | 0.002 |
| m6_1 | 2 | 8 | 16.651 | 0.002 | 48.651 | 12.240 | 0.001 |
| m3_3 | 3 | 7 | 23.766 | 0.001 | 48.966 | 12.555 | 0.001 |
| m2_5 | 2 | 8 | 17.528 | 0.002 | 49.528 | 13.117 | 0.001 |
| m7_5 | 2 | 8 | 17.528 | 0.002 | 49.528 | 13.117 | 0.001 |
| m5_2 | 3 | 7 | 25.985 | 0.000 | 51.185 | 14.774 | 0.000 |
| m2_3 | 3 | 7 | 26.379 | 0.000 | 51.579 | 15.168 | 0.000 |
| m7_3 | 3 | 7 | 26.379 | 0.000 | 51.579 | 15.168 | 0.000 |
| m2_1 | 3 | 7 | 28.246 | 0.000 | 53.446 | 17.034 | 0.000 |
| m5_1 | 3 | 7 | 28.592 | 0.000 | 53.792 | 17.381 | 0.000 |
| m7_1 | 3 | 7 | 28.796 | 0.000 | 53.996 | 17.584 | 0.000 |
| m1_6 | 1 | 9 | 13.718 | 0.001 | 54.218 | 17.807 | 0.000 |
| m4_6 | 1 | 9 | 13.718 | 0.001 | 54.218 | 17.807 | 0.000 |
| m6_6 | 1 | 9 | 13.718 | 0.001 | 54.218 | 17.807 | 0.000 |
| m4_2 | 2 | 8 | 23.168 | 0.000 | 55.168 | 18.756 | 0.000 |
| m6_2 | 2 | 8 | 23.168 | 0.000 | 55.168 | 18.756 | 0.000 |
| m3_6 | 2 | 8 | 23.250 | 0.000 | 55.250 | 18.839 | 0.000 |
| m2_6 | 2 | 8 | 25.863 | 0.000 | 57.863 | 21.452 | 0.000 |
| m7_6 | 2 | 8 | 25.863 | 0.000 | 57.863 | 21.452 | 0.000 |
| m1_2 | 2 | 8 | 26.332 | 0.000 | 58.332 | 21.921 | 0.000 |
| m3_1 | 3 | 7 | 35.306 | 0.000 | 60.506 | 24.095 | 0.000 |
| m3_2 | 3 | 7 | 35.864 | 0.000 | 61.064 | 24.653 | 0.000 |
| m2_2 | 3 | 7 | 46.333 | 0.000 | 71.533 | 35.121 | 0.000 |
| m7_2 | 3 | 7 | 46.333 | 0.000 | 71.533 | 35.121 | 0.000 |
| k= number of independence claims; q= number of parameters | | | | | | | |

**Table S3.**

Effect of brain mass (residual) and max adult life span (max life span - AFR) on **a**) S_A_ (survival rate until female AFR), and **b**) S_1_ (survival rate until age 1).

|  | Estimate | se | t | p |
| --- | --- | --- | --- | --- |
| **a) Survival rate until AFR (**S_A_**) with absolute brain size** | | | | |
| Intercept | -0.194 | 0.508 | -0.382 | 0.708 |
| **Brain mass (residual)** | 0.587 | 0.226 | 2.593 | **0.020*** |
| **max adult life span** **(log)** | 0.075 | 0.210 | 0.357 | 0.726 |
| **b) Survival rate until age 1 (**S_1_**) with absolute brain size** | | | | |
| Intercept | -0.151 | 0.569 | -0.266 | 0.794 |
| **Brain mass (residual)** | 0.777 | 0.275 | 2.831 | **0.013*** |
| **max adult life span** **(log)** | -0.254 | 0.257 | -0.985 | 0.340 |
| Maximum adult lifespan (in years) was primarily obtained from Isler and van Schaik (2012; DOI: 10.1016/j.jhevol.2012.03.009), except for *Gorilla beringei*, which was sourced from Myhrvold et al. (2015; DOI: 10.1890/15-0846R.1). | | | | |

**Figure**

**Figure S1**

**Figure S1.** 42 candidate path models were tested using phylogenetic path analysis to explore the relationships among body size, brain size, age at first reproduction (AFR), and survival until AFR (Surv_AFR). Models were evaluated based on d-separation tests, with overall fit quantified using Fisher’s C statistic and ranked using CICc.
